# Supplementary material for: A Role for the Interactions between Polδ and PCNA Revealed by Analysis of pol3-01 Yeast Mutants
Source: Genes (Basel). 2023 Feb 2;14(2):391. doi: 10.3390/genes14020391 (PMC9957047; doi:10.3390/genes14020391)
Supplement: Supplementary file 1 [file genes-14-00391-s001.zip › genes-2196430-supplementary.pdf]

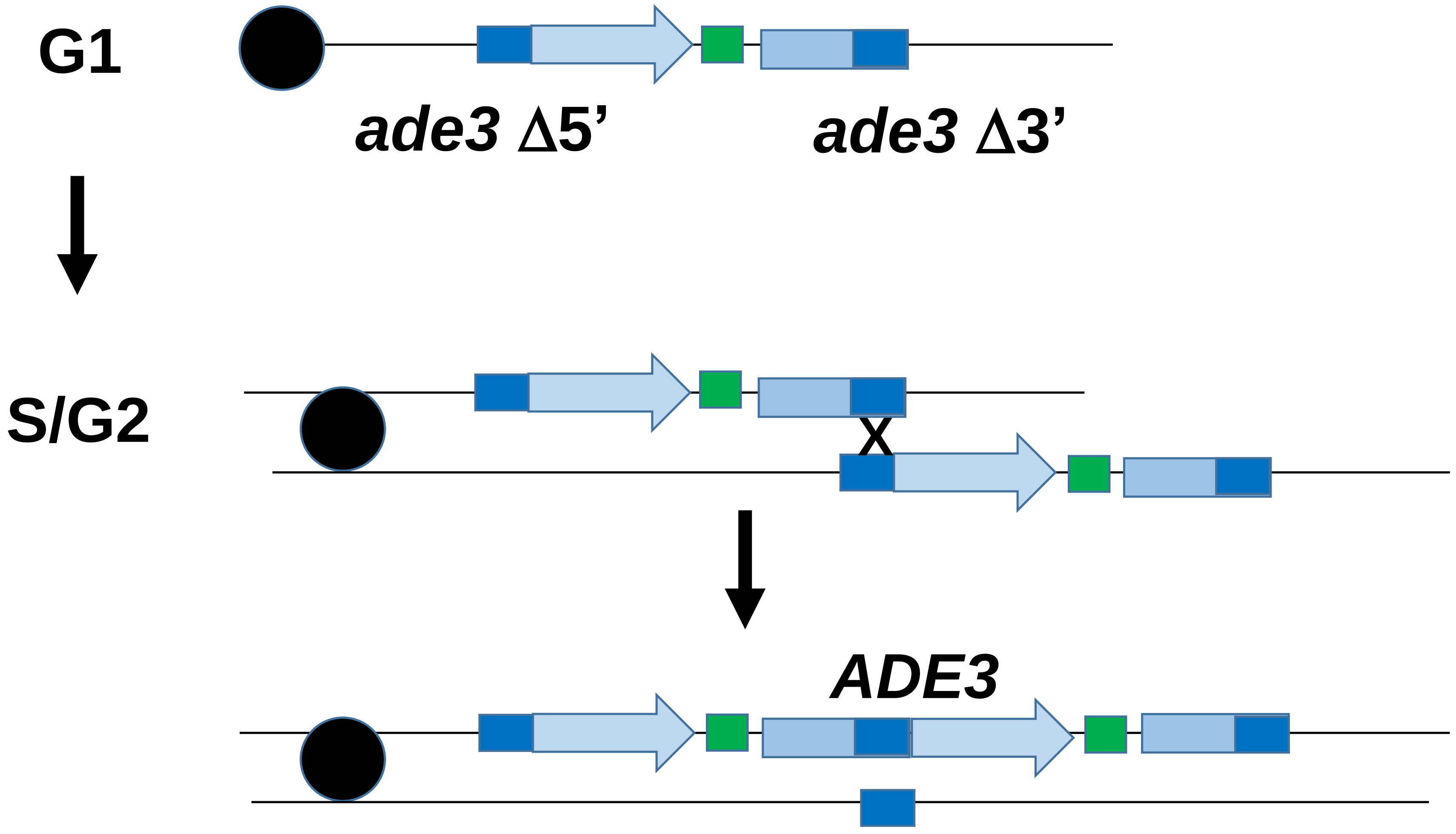

Figure S1

Figure S1: Assay for measuring Unequal Sister Chromatid Recombination (USCR) : Strain BLS2 contains at its chromosome III, 5’ *ade3* and 3’ *ade3* fragments that are separated by *URA3* marker. This construct allows to detect new USCR by plating the cells on the appropriate medium (SD-HIS or SD-ADE). In wild type cells, HIS+ appear in the wild type at a rate of $5\text{-}6x10^{-5}$.

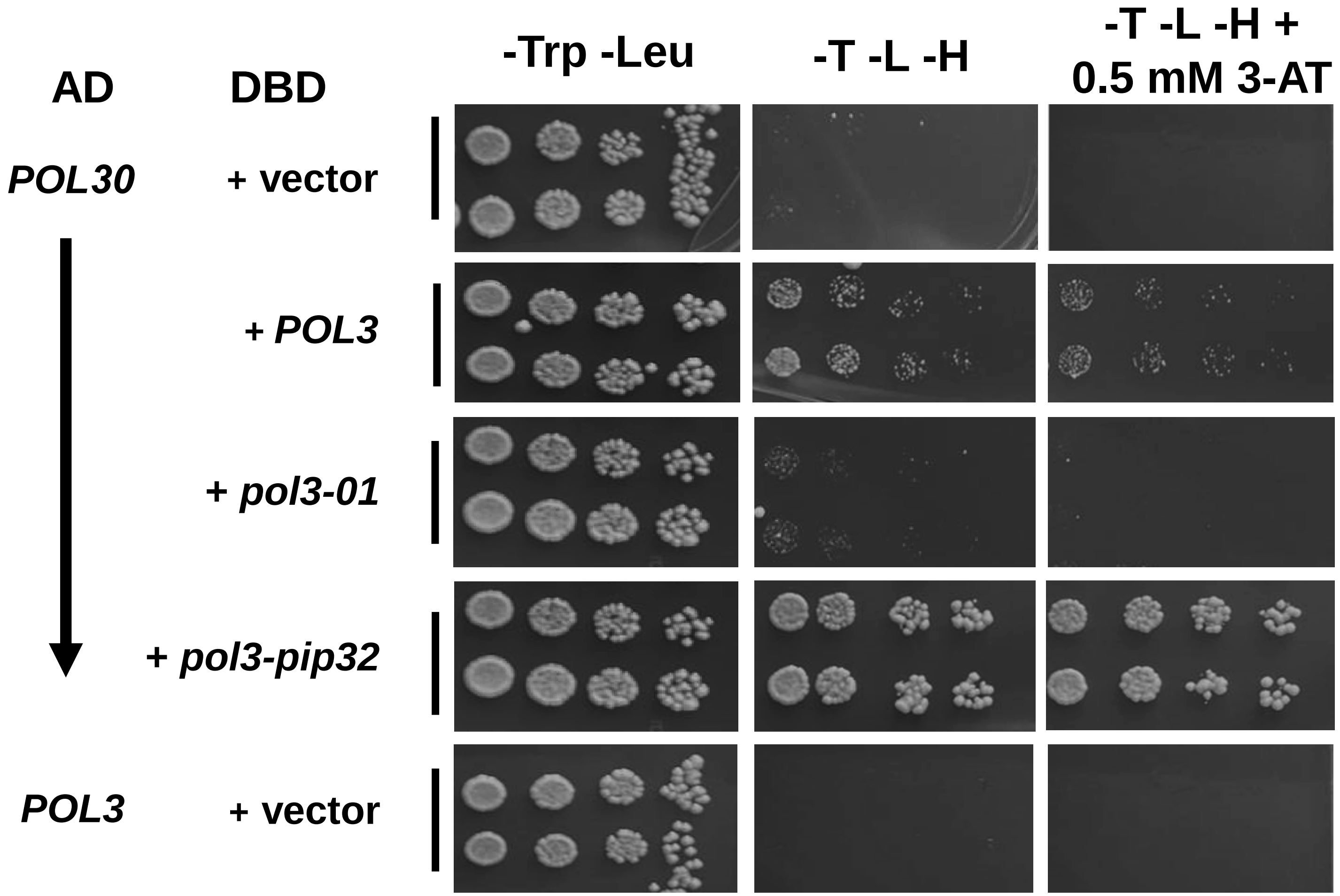

AD
DBD
-Trp -Leu
-T -L -H
-T -L -H +
0.5 mM 3-AT
POL30
+ vector
+ POL3
+ pol3-01
+ pol3-pip32
POL3
+ vector

Figure S2

Figure S2: Yeast Two Hybrid experiment to test interactions between POL30 (PCNA) fused to the activating domain (AD)of Gal4, and different *POL3* alleles fused to the DNA Binding domain of Gal4 (DBD).
